# Glucagon-Like Peptide-1 Receptor Agonist Mediated Weight Loss and Diabetes Mellitus Benefits: A Narrative Review

**DOI:** 10.7759/cureus.76101

**Published:** 2024-12-20

**Authors:** Alan D Kaye, Nathan Lien, Christopher Vuong, Matthew H Schmitt, Yusra Soorya, Bushirat A Abubakar, Luke Muiznieks, Noah Embry, Harish Siddaiah, Adam M Kaye, Sahar Shekoohi, Giustino Varrassi

**Affiliations:** 1 Department of Anesthesiology, Louisiana State University Health Sciences Center, Shreveport, USA; 2 School of Medicine, Louisiana State University Health Sciences Center, Shreveport, USA; 3 School of Medicine, Louisiana State University Health Sciences Center, New Orleans, USA; 4 Department of Emergency Medicine, Louisiana State University Health Sciences Center, Shreveport, USA; 5 Department of Pharmacy Practice, Thomas J. Long School of Pharmacy and Health Sciences, University of the Pacific, Stockton, USA; 6 Department of Pain Medicine, Fondazione Paolo Procacci, Rome, ITA

**Keywords:** diabetes treatment, glp-1, glucagon-like peptide-1 receptor agonist, obesity management, weight loss

## Abstract

Obesity and type 2 diabetes mellitus (T2DM) are chronic diseases with increasing prevalence, underscoring the urgent need for effective treatment and management strategies. Glucagon-like peptide-1 receptor agonists (GLP-1 RAs) have emerged as an essential class of drugs for managing both obesity and T2DM, offering additional benefits for cardiovascular and kidney health. GLP-1 RAs work by targeting GLP-1 receptors, mimicking the effects of the natural hormone GLP-1 to regulate blood glucose levels, promote weight loss, and provide potential benefits for cardiovascular health. This narrative review evaluates the mechanisms of action, clinical efficacy, and broader roles of GLP-1 RAs in promoting weight loss and glycemic control. In addition, the present investigation explores recent clinical studies demonstrating the effectiveness of GLP-1 RAs in diabetic and nondiabetic populations, highlighting their potential in addressing obesity even in those without T2DM and describing probable benefits to cardiovascular health. Finally, our investigation outlines the importance of future research to further define the potential benefits of GLP-1 RAs.

## Introduction and background

In recent years, glucagon-like peptide-1 receptor agonists (GLP-1 RAs) have gained significant attention as effective treatments for type 2 diabetes mellitus (T2DM) and obesity, with emerging potential in renal, pulmonary, and other therapeutic areas. These drugs mimic the effects of the hormone glucagon-like peptide-1 (GLP-1), which is primarily involved in glucose metabolism. Released from the intestines after food intake, GLP-1 stimulates insulin secretion, inhibits glucagon release, and slows gastric emptying, thereby reducing postprandial blood sugar levels [1]. In addition, it also influences appetite regulation, making it beneficial for weight management, which is a crucial factor in managing diabetes and metabolic syndrome. The popularity of GLP-1 RAs has surged due to their ability to lower hemoglobin A1C (HbA1c) in people with T2DM while promoting weight loss. Medications like liraglutide, semaglutide, and dulaglutide are now widely prescribed because they offer advantages over older diabetes medications that often cause hypoglycemia or weight gain. GLP-1 RAs are generally associated with minimal hypoglycemia risk, making them a safer alternative for blood sugar management [1]. Furthermore, research demonstrates their cardiovascular benefits, including reduced major adverse cardiovascular events, further driving their adoption [1]. These advantages have expanded their usage beyond diabetes management to obesity treatment, earning widespread acceptance among patients and healthcare providers seeking comprehensive strategies for diabetes care and weight reduction.   

In addition, the prevalence of obesity has steadily increased over the past decades, contributing to higher rates of morbidity and disability across populations. Obesity is characterized by excessive fat accumulation resulting from genetic, environmental, or other factors. Common interventions for weight loss include diet, exercise, prescription medications, and bariatric surgery [2]. Among these, GLP-1 RAs have emerged as a promising treatment option for aiding weight loss in obese populations. Therefore, this narrative review aims to discuss the potential benefits and effects of GLP-1 agonists in managing diabetes and obesity. It also highlights their potential for broader applications in medicine, emphasizing their versatility as therapeutic agents. Specifically, this investigation examines their role in weight reduction, diabetes management, cardiovascular health, and prospective future applications.

## Review

The role of GLP-1 agonist in weight loss: mechanism of actions and clinical outcomes

Mechanism of Action

In discussing the mechanism of action of GLP-1 RAs, it is important to consider the historical context of these medications. Exenatide, a synthetic protein derived from the saliva of the Gila monster, has played a pivotal role in advancing treatments for diabetes mellitus (DM) and weight management [3]. The Gila monster’s infrequent eating habits, where it only consumes food once or twice a year, captured the interest of gastroenterologist Jean-Pierre Raufman. He discovered that the lizard’s saliva contains biologically active molecules capable of inducing pancreatic inflammation in test animals [4]. This discovery prompted research into its potential for DM treatment and appetite reduction, ultimately leading to the development of exenatide as a GLP-1 receptor agonist [3,4]. Exogenous GLP-1 receptor agonists, including exenatide, liraglutide, and semaglutide, function by mimicking the incretin hormone, GLP-1, to stimulate insulin secretion in T2DM, where endogenous incretin function and insulin sensitivity are impaired. These agonists help restore insulin responsiveness by enhancing the incretin effect. Unlike endogenous GLP-1, these agents are resistant to degradation by dipeptidyl peptidase-4 (DPP-4), extending their half-life and enabling sustained action [5]. ​The pharmacokinetics of GLP-1 RAs exhibit distinct characteristics. Exenatide is available in immediate-release (twice daily) and extended-release (weekly) formulations, with elimination primarily via glomerular filtration followed by proteolytic degradation. Liraglutide, a daily injectable, is metabolized by DPP-4 and endogenous endopeptidases, enhancing its efficacy for weight loss and glycemic control, and can be combined with insulin degludec for improved management. Semaglutide is administered as either a weekly injection or daily oral tablet and is metabolized into amino acids by serum and tissue proteases, which support its prolonged action. Lastly, dulaglutide, a large fusion protein given weekly, has an extended half-life due to resistance to DPP-4 degradation and is primarily catabolized through general protein degradation rather than cytochrome P450 pathways [1]. ​The pharmacodynamic properties of these agents, such as varied half-lives and targeted effects on glucose-regulating tissue, make them versatile in managing T2DM by addressing both fasting and postprandial glucose levels, improving cardiovascular health, and supporting weight reduction efforts. Their limited reliance on cytochrome P450 metabolism reduces the risk of drug-drug interactions, making them suitable for patients on multiple medications [5].​ This diverse array of mechanisms underscores the clinical value of exogenous GLP-1 agonists as an effective and multifaceted therapeutic option for managing T2DM.  

GLP-1 Impact on Weight Reduction

Obesity is associated with a range of adverse health conditions, including cardiovascular disease, chronic kidney disease, and even cancer, due to the increased body fat percentages. Potential treatments for obesity are medications containing GLP-1 RAs. While GLP-1 injections are often used to achieve normoglycemia in T2DM, some studies have also demonstrated their significant efficacy in promoting weight loss [6]. In a phase 3, double-blind, randomized study, 2539 adults with a BMI ≥ 30 but no diagnosis of diabetes were given tirzepatide, a dual-action gastric inhibitory peptide (GIP), and GLP-1 RAs. Participants received either tirzepatide or a placebo at a 2.5-mg dose once weekly during the study (72 weeks), with a dosage escalation of 2.5 mg every four weeks. In the study's conclusion, tirzepatide significantly outperformed the placebo in promoting weight loss. Participants administered 5 mg, 10 mg, and 15 mg of tirzepatide achieved mean weight losses of 15.0%, 19.5%, and 20.9%, respectively, while the placebo group had a mean weight loss of only 3.1%. Notably, most prediabetic participants who received tirzepatide for the trial reverted to a normoglycemic state [7]. The results indicated the effectiveness of tirzepatide in promoting weight loss among the participants, likely due to its ability to target multiple hormone pathways involved in energy balance. The highest weight loss outcomes were observed at the 15-mg dose. However, it was noted that participants taking the higher dosage were reported to have gastrointestinal side effects. Specifically, participants treated with tirzepatide reported higher rates of adverse effects such as nausea, diarrhea, and constipation compared to the placebo group, with nausea rates of 18.7%, 22.7%, and 25.6% for 5-mg, 10-mg, and 15-mg tirzepatide doses, respectively, compared to 9.5% in the placebo group [7]. These adverse events were most often reported as mild to moderate in severity and occurred predominately during dose escalation. The study indicated that further research is needed to determine long-term effects in a larger population.  

In a randomized, double-blind, Semaglutide Effects on Cardiovascular Outcomes in People with Overweight or Obesity (SELECT) study, 17,604 obese participants with established cardiovascular disease without a diabetes diagnosis were given semaglutide or a placebo. Semaglutide was included in this study due to its proven reduction in major cardiovascular events and its potential as an effective weight loss aid. The participants were subcutaneously injected with 2.4 mg of semaglutide or placebo once weekly over the course of the study. The results showed that semaglutide participants had an average weight loss of 10.2% over a four-year period, while the placebo group had an average weight loss of 1.5% [6]. Semaglutide was found to have significant results in this study, not only for lowering weight but also for reducing adverse cardiovascular events. An alternative study focused on the effects of liraglutide on the treatment of obesity. The 564 obese participants in the randomized, double-blind study were divided into two groups: those who received liraglutide and those who received a placebo for a 20-week period. They received a placebo or differing doses of liraglutide (1.2 mg, 1.8 mg, 2.4 mg, or 3.0 mg) which were injected subcutaneously. It is important to note that participants were encouraged to increase their physical activity through two-week run-ins and that a calorie deficit of 500 calories was implemented. The results of the study showed that participants experienced significant weight loss when taking liraglutide compared to the placebo. The average weight loss with liraglutide at doses of 1.2, 1.8, 2.4, and 3.0 mg was 4.8, 5.5, 6.3, and 7.2 kg, respectively, compared to 2.8 kg weight loss with the placebo. However, participants taking liraglutide did experience symptoms of nausea and vomiting compared to those taking a placebo [8]. The study showed that combining GLP-1 RAs with a calorie deficit and/or increased physical activity has synergistic effects on weight loss in obese participants compared to a placebo. 

All the studies mentioned have shown significant results in the use of GLP-1 subcutaneous injections to lower the BMI of obese participants compared to those taking placebo. GLP-1 RAs medications with gradual and prolonged use are shown to reduce weight and maintain weight at those lowered levels [6]. Additionally, the use of GLP-1 RAs in conjunction with increased physical activity and a caloric deficit shows significant weight loss [8]. However, some participants receiving GLP-1 RAs at higher doses were shown to have adverse symptoms like nausea and vomiting [7,8]. In conclusion, participants with underlying conditions like prediabetes and cardiovascular disease can use differing types of GLP-1 therapies that cater more toward prediabetic therapy and reduce the risk of cardiovascular disease, respectively (Table 1).

**Table 1:** Functional profile of GLP-1 RAs T2DM: type 2 diabetes mellitus.

GLP-1 RAs	Receptors	Function	Dosage
Liraglutide [9]	Glucagon-like peptide-1	T2DM and reducing the risk of major adverse cardiovascular events	0.6 mg/mL, 1.2 mg/mL, 1.8 mg/mL, 2.4 mg/mL, 3 mg/mL
Semaglutide [10]	Glucagon-like peptide-1	T2DM and reducing the risk of major adverse cardiovascular events	0.25 mg/0.5 mL, 0.5 mg/0.5 mL, 1 mg/0.5 mL, 1.7 mg/0.75 mL, 2.4 mg/0.75 mL
Dulaglutide [11]	Glucagon-like peptide-1	T2DM and reducing the risk of major adverse cardiovascular events	0.75 mg/0.5 mL, 1.5 mg/0.5 mL, 3 mg/0.5 mL, 4.5 mg/0.5 mL
Tirzepatide [12]	Glucagon-like peptide-1 glucose-dependent insulinotropic	T2DM and reducing the risk of major adverse cardiovascular events	2.5 mg/0.5 mL, 5 mg/0.5 mL, 7.5 mg/0.5 mL, 10 mg/0.5 mL, 12.5 mg/0.5 mL, 15 mg/0.5 mL

GLP-1's impact on managing DM

GLP-1 RAs have been classically used for DM prevention and treatment. DM affects nearly half a billion people worldwide, 463 million of whom have T2DM [13]. People with T2DM are at increased risk for metabolic syndrome, cardiovascular disease, and kidney disease, which reduces life expectancy and quality of life. GLP-1 stimulates insulin release from β-cells in the islets of Langerhans and consequently has classical insulinotropic effects, such as slowing gastric emptying and enhanced fasting [14]. Therefore, in addition to their anti-obesity effects, GLP-1 RAs can be used for T2DM related to insulin-independent glucose-lowering effects [15]. In addition to the primary treatment of T2DM, GLP-1 RAs have been found to reduce adverse cardiovascular and renal events without an increase in pancreatic cancer or pancreatitis [16]. The American Diabetes Association supports the use of GLP-1 RAs for DM specifically related to a lower risk of hypoglycemia in comparison with basal insulin [17]. There is evidence, however, that GLP-1 RAs use can be added to metformin, a traditional diabetes treatment, to treat hyperglycemia in T2DM [18]. GLP-1 RAs are often grouped with SGLT-2 inhibitors, which prevent glucose reuptake in the kidney, as novel therapeutic approaches for the treatment of cardiovascular and renal disease in T2DM, but more research is needed for systematic differences in benefits and harms between them [19]. However, T2DM treatment is nuanced, with therapeutic challenges for clinicians and patients. Pharmacotherapy must be initiated based on expected longevity, the presence of diabetic complications, and patient preferences [17]. Patients taking GLP-1 RAs suffer from increased adverse effects compared to placebo, including nausea and vomiting and especially weight loss, which may be desirable in some patients and undesirable for others [20]. Clinicians must be careful in the concomitant prescription of multiple insulinotropic medications, and dosages may have to be adjusted to reduce hypoglycemic events [21]. In conclusion, GLP-1 RAs are effective in treating T2DM compared to both placebo and metformin, but care must be taken in managing side effects and adverse drug effects.

GLP-1's role in cardiovascular health and potential

DM and its role in accelerated atherosclerosis with increased risk for cardiovascular disease (CVD) have been well-established in scientific literature. Patients with DM can develop CVD, dependent on a wide array of factors, e.g., hypertension, elevated LDL cholesterol, and HbA1C levels [22]. Patients with DM who develop CVD have a two- to fourfold higher mortality rate than patients without DM [23]. CVD is the most prevalent cause of mortality in diabetic patients [24]. Therefore, treatment with GLP-1 RAs is an essential area of research to prevent cardiovascular complications in patients with DM. One of the most critical factors for the development of CVD is the development of atherosclerosis, termed the “invisible killer” [25]. In patients with T2DM, HbA1C, dyslipidemia, blood pressure (BP), and body weight are some of the most critical risk factors for the development of atherosclerosis [25]. Atherosclerosis is a chronic inflammatory condition defined by the deposition of cholesterol-rich lipoproteins into the arterial cell walls, further promoting inflammatory responses that accelerate atherosclerosis [26]. GLP-1 RAs have been shown to reduce the formation of these atherosclerotic plaques and reduce the production of TNF-a and interleukin-1 (IL-1), two proinflammatory cytokines [27]. In one study, ApoE−/− and LDLr−/− mice were given semaglutide and liraglutide, a condition that significantly decreased plaque lesion development and reduced inflammatory pathogenesis [28]. Mice receiving liraglutide displayed a decrease in aortic plaque area to 18.8 ± 1.5%, compared to the control group with 25.3 ± 2.2% [28]. In the case of semaglutide, all dose levels significantly reduced aortic plaque area, with the intermediate dose of 12 μg/kg providing the greatest reduction (4.0% ± 0.8%) [28]. The low (4 μg/kg) and high (60 μg/kg) doses also resulted in significant reductions in plaque area (4.5% ± 0.8% and 4.6% ± 1.1%, respectively), which were all significantly lower than the control (13.1% ± 1.3%) [28]. This study demonstrated that GLP-1 medications can benefit cardiovascular health by limiting the progression of atherosclerotic plaque development in mice. 

Patients with T2DM are also at an enhanced risk for developing chronic kidney disease (CKD), defined by a decrease in glomerular filtration rate (GFR) from progressive damage to the structure and function of the kidney, and increased proteinuria [25]. This damage to the kidney is mediated by poor glycemic control, as blood glucose control is one of the most important interventions to prevent diabetic CKD [29]. GLP-1RAs prove to be effective at lowering blood glucose, thus providing beneficial effects at lowering kidney damage, but the mechanism is not entirely understood [29]. However, some proposed mechanisms include reducing inflammation, weight loss, and increasing sodium excretion in the kidneys by blocking sodium/hydrogen isoform 3 (NHE3) [30]. In the SUSTAIN-6 trial, only 3.8% of diabetic patients receiving semaglutide experienced new or worsening nephropathy, compared to 6.1% in the placebo group [31]. This data suggests that GLP-1 RAs have a beneficial effect on kidney function in diabetic patients. As stated previously, diabetes increases the risk of developing CVD. Diabetic cardiomyopathy is a vital complication arising from downstream effects of chronic hyperglycemia, such as oxidative stress, fibrosis of the myocardium, impaired myocyte calcium metabolism, and microangiopathy [32]. This condition is an important risk factor for developing heart failure (HF), with men being twice as likely and women being five times as likely to develop HF compared to patients without diabetes [33]. GLP-1 RAs have been shown to relieve myocardial damage by promoting autophagy, a process that aids in maintaining homeostasis in response to unfavorable cellular environments such as hyperglycemia and oxidative stress, both seen in diabetes [34]. Additionally, GLP-1 RAs have been shown to reduce apoptosis in cardiomyocytes experiencing these stressors by inhibiting the receptor of advanced glycation end products (RAGE) [35]. In this study, cardiac myocytes exposed to high levels of glucose experienced a time-dependent increased RAGE expression, with peak expression at 24 hours [35]. When these cells were exposed to exendin-4 (EX-4), RAGE expression decreased and thus provided a cardioprotective effect [35]. These studies and others have demonstrated promising benefits for reducing HF in diabetic patients with GLP-1 RAs [36]. Additionally, in one recent study, it was noted that semaglutide was used in patients with heart failure with preserved ejection fraction and obesity and was seen to lead to a reduction in symptoms, improved exercise function, and weight loss [37]. GLP-1 RAs have been shown to benefit multiple health factors, including decreasing atherosclerotic plaque development, decreasing cardiac inflammation, and improving kidney function.

Discussion

Within this review, various aspects of GLP-1 RAs have been discussed, including their efficacy, mechanism of action, role in T2DM management, and cardiovascular protective effects. GLP-1 RAs have transformed the management of obesity and T2DM, primarily due to their ability to regulate blood glucose levels and induce significant weight loss. For patients with obesity, GLP-1 RAs have emerged as a noninvasive alternative to bariatric surgery, offering compelling weight loss with similar effects in glycemic control [36]. This makes GLP-1 RAs an appealing option for individuals who may not be candidates for surgical interventions, while still offering a safe and accessible treatment alternative. Besides their action on weight loss, GLP-1 RAs represent a valuable option for managing T2DM, particularly when compared to other classic options, such as SGLT2 inhibitors. Notably, research has demonstrated that GLP-1 RAs not only effectively control blood glucose levels but also provide added benefits in terms of reduced risk of cardiovascular events when compared to other agents such as SGLT2 inhibitors [38]. The ability to simply serve as an effective alternative is compelling on its own, with the added benefit of offering more protective effects than other traditional options.

Additionally, recent research has explored the possibility of using GLP-1 RSs in treating and slowing the progression of adverse kidney events. In one study, for example, the renal protective effects of GLP-1 RAs were examined in those with advanced diabetic kidney disease [39]. The study found that GLP-1 RAs exhibited significant renal protective effects and were associated with reduced composite kidney effects, including a decline in the estimated glomerular filtration rate and slowed progression of end-stage kidney disease when compared to patients on dipeptidyl peptidase 4 inhibitors [39].

Despite these promising results, limitations exist in the studies reviewed. Many trials focused on short-term outcomes and did not evaluate the long-term efficacy and safety of GLP-1 RAs. Some of the studies also primarily included individuals with T2DM or obesity, which limits the generalizability of the findings to broader populations. Furthermore, the adverse effects of GLP-1 RAs, particularly severe ones such as gastrointestinal issues and pancreatitis, are an area that needs to be explored. Given this, the therapeutic potential of GLP-1 RAs is seemingly endless and largely unexplored. Future research will be essential to fully elucidate the long-term efficacy and safety of GLP-1 RAs. Studies comparing various GLP-1 RAs, similar to the direct comparison between tripeptide and semaglutide, could provide more insight into which formulation or dose offers the most favorable outcomes in terms of weight loss and glycemic control [40]. Additionally, exploring the interactions of GLP-1 RAs with other commonly prescribed medications for other comorbid conditions, such as fibrates or antihypertensive agents, could reveal how these drugs may work synergistically to outcomes in patients. Lastly, future research might also investigate the potential of GLP-1 RAs in treating other diseases beyond T2DM and obesity. Recent studies have illustrated how GLP-1 RAs may offer potential benefits in treating fatty liver disease and polycystic ovary syndrome [41,42]. Both of these conditions are common comorbidities that are associated with metabolic dysfunction. With their ever-increasing range of therapeutic potential, the continual development and use of GLP-1 RAs seems inevitable. Further research into these agents is necessary to fully uncover their benefits and to discover their role in treating other conditions. 

## Conclusions

GLP-1 RAs are a crucial and rapidly advancing class of drugs, quickly becoming a popular mediator in the treatment of both obesity and T2DM. This narrative review discusses the mechanisms of key GLP-1 RAs, including exenatide, liraglutide, and semaglutide, and examines various clinical trial data supporting their effectiveness in achieving substantial weight reduction in individuals with and without diabetes. Additionally, we explored the role of GLP-1 RAs in T2DM management, including their ability to improve fasting glucose control and slow gastric emptying. Finally, the role of GLP-1 RAs in cardiovascular protection was also highlighted, and their role was focused on decreasing the formation of atherosclerotic plaques and alleviating myocardial damage. While GLP-1 RAs are a very promising class of drugs, they are still relatively new, and further research is essential to expand on their therapeutic potential. Research into areas such as examining the long-term effects, understanding potential applications, and optimizing dosing strategies is needed to ensure the continued safety of these medications. Overall, however, this narrative review highlights the vast potential of GLP-1 RAs and provides a foundation for additional research to refine and broaden current treatment protocols. As obesity and T2DM continue to evolve and increase in prevalence, GLP-1 RAs' role in managing these chronic diseases will continue to expand. As a result, research must continue to improve these agents and enhance their role in chronic disease management.
